# Rare and Fascinating Case of ST-Elevation Myocardial Infarction Diagnosis From an Underlying Ventricular Paced Rhythm

**DOI:** 10.7759/cureus.8274

**Published:** 2020-05-25

**Authors:** Woosun Kang, Liang D Ge, Puja Patel, Raj Patel, Tinoy Kizhakekuttu

**Affiliations:** 1 Internal Medicine, University of Illinois College of Medicine at Peoria, Peoria, USA; 2 Internal Medicine, American University of Antigua, Peoria, USA; 3 Cardiology, University of Illinois College of Medicine at Peoria, Peoria, USA

**Keywords:** ventricular paced rhythm, electrophysiology, cardiac catheterization, acute coronary syndrome, cardiology, cardiology research

## Abstract

This is a case of a patient diagnosed with anterior ST-elevation myocardial infarction (STEMI) with a ventricular paced rhythm after the patient underwent a femoral-femoral bypass surgery for severe peripheral vascular disease. The case highlights the diagnosis of STEMI in the setting of paced rhythm in the appropriate clinical setting.

## Introduction

It is difficult to diagnose ST-elevation myocardial infarction (STEMI) in the setting of an abnormal baseline EKG, such as a left bundle branch block (LBBB). Fortunately, the Sgarbossa criteria have been accepted as a highly specific (96%) algorithm for the diagnosis of STEMI with concurrent LBBB [1]. Patients who are paced in the right ventricle manifest iatrogenic LBBB. Thus, the Sgarbossa criteria can be used as a tool for STEMI diagnosis in the setting of right ventricular paced rhythm (VPR) [2-4]. This case highlights the clinical decision-making for the diagnosis of STEMI in a patient with VPR after recent femoral-femoral bypass surgery for severe peripheral vascular disease.

## Case presentation

**Figure 1:**
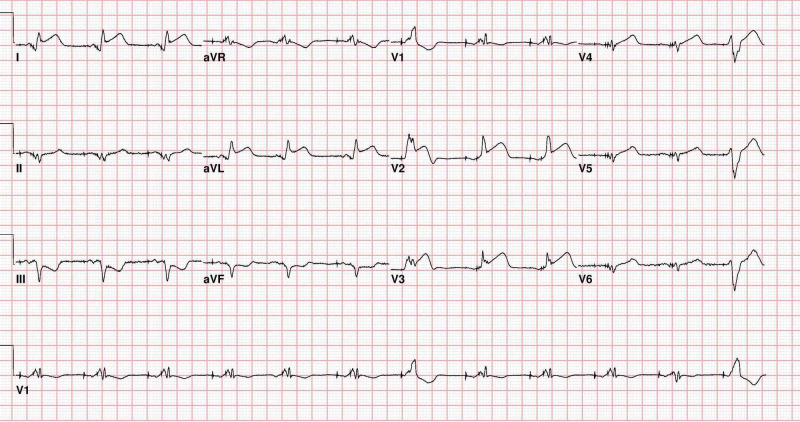
Presenting EKG revealing ventricular paced rhythm with ST elevations in the anterior and lateral leads with inferior depression.

**Figure 2:**
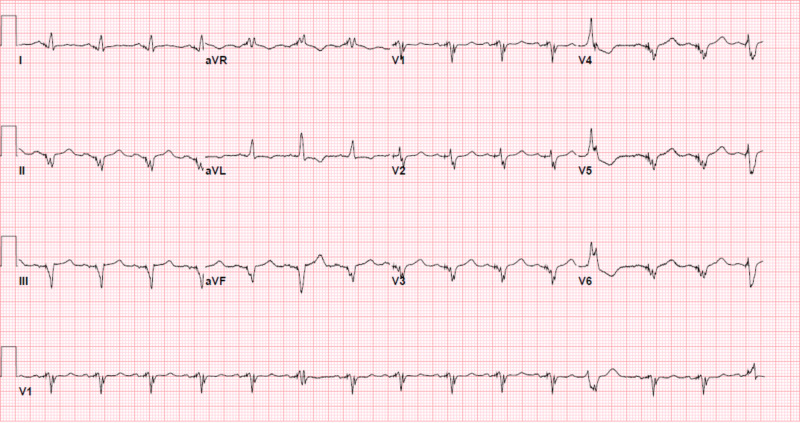
Baseline EKG several months prior to presentation for STEMI. STEMI, ST-elevation myocardial infarction

This is the case of an 83-year-old male with a medical history of essential hypertension, hyperlipidemia, ischemic cardiomyopathy, coronary artery disease (CAD), PVD, and abdominal aortic aneurysm. The patient has a known history of bi-ventricular implantable cardioverter defibrillator implantation and aortic aneurysm repair. She presented to the emergency department with typical angina. Two weeks prior to presentation, the patient was discharged after femoral-femoral bypass surgery for severe lower extremity PVD. Presenting EKG revealed concordant > 1 mm ST-segment elevations in the anterior and inferior leads (Figure 1), which were new compared to baseline EKG (Figure 2). Initial vitals were as follows: temperature of 98.2°F, pulse rate of 74, blood pressure of 130/76, respiratory rate of 20 breaths per minute, and SpO_2_ of 97% on room air. Physical examination revealed bilateral lung rales and 1+ bilateral lower extremity edema. No jugular venous distension or carotid bruits were noted and peripheral pulses were symmetric, 2+, and intact. Initial troponin was elevated at 0.111. Urgent transthoracic echocardiogram (TTE) showed visually decreased left ventricular ejection fraction (LVEF) of 30-35%, which was reduced from a baseline of 40-45%. Additionally, basal-to-inferior and mid-inferior wall akinesis was noted, as well as severely hypokinetic mid-to-apical anterior and anteroseptal walls.

**Figure 3:**
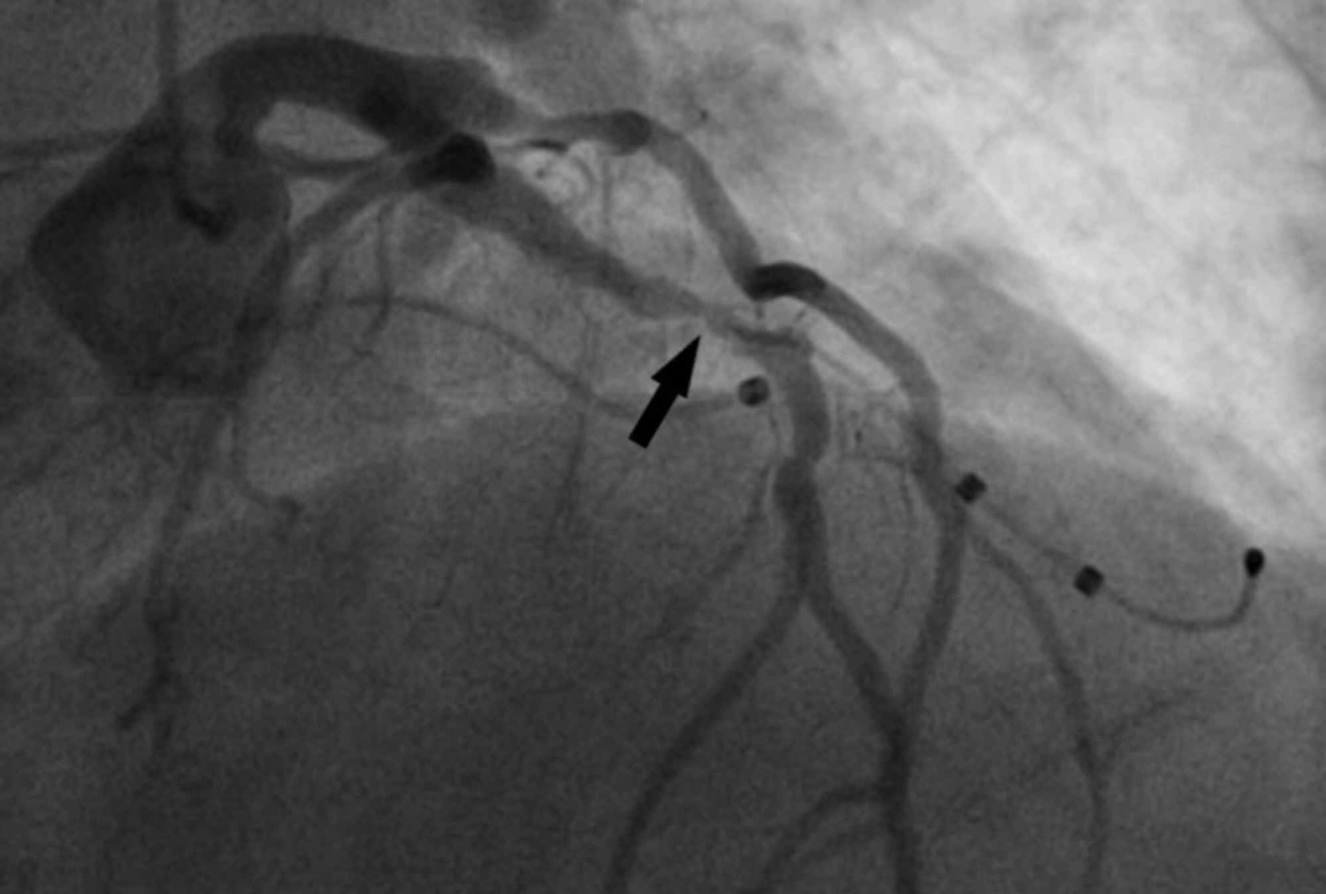
Severe proximal left anterior descending artery stenosis with an acute, ulcerated appearance.

**Figure 4:**
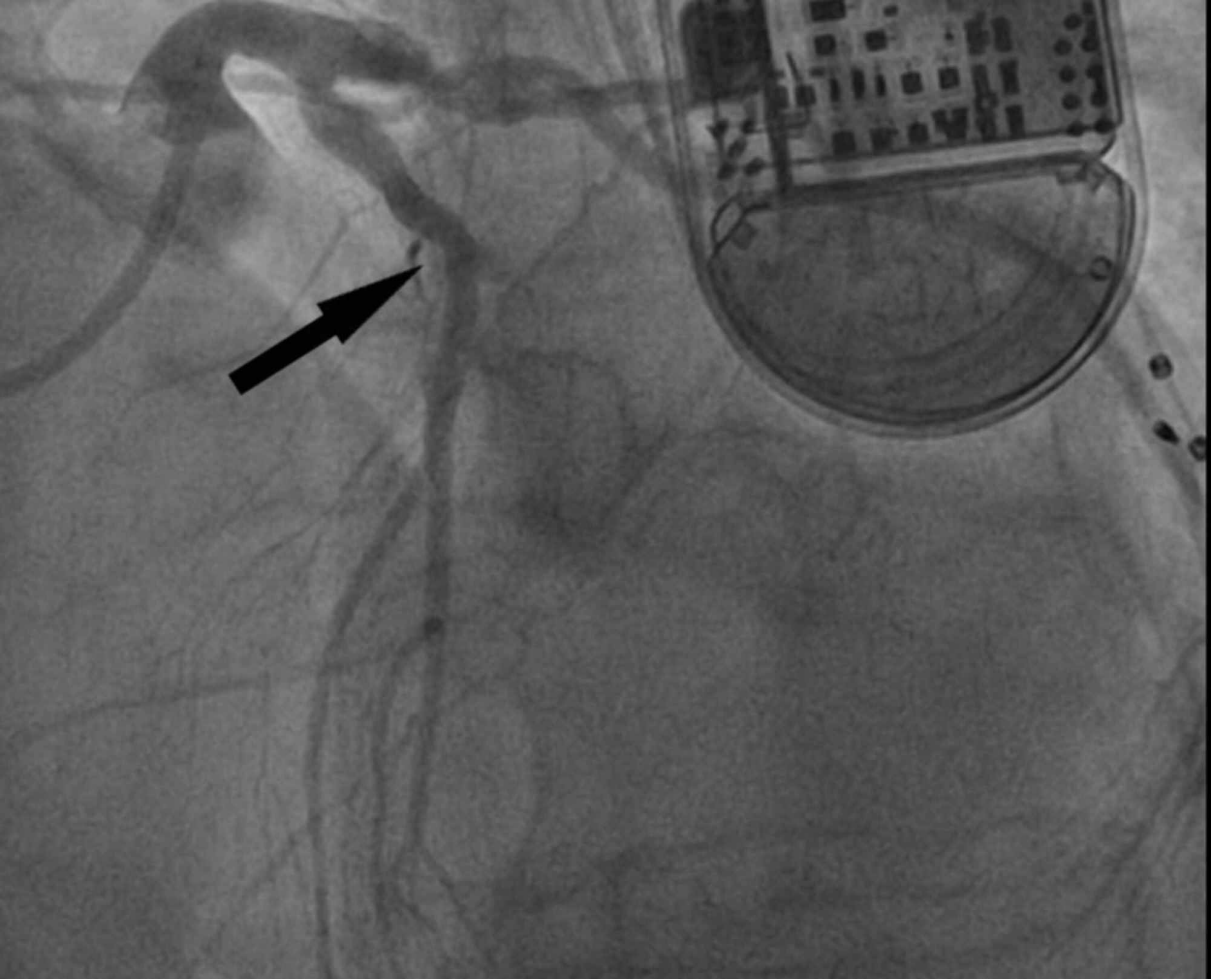
Arrow showing a successful PCI with a drug-eluting stent to the proximal left anterior descending artery. PCI, percutaneous coronary intervention

HHeparin drip was initiated in the emergency room, and the patient was taken for emergent cardiac catheterization/coronary angiography. A loading dose of ticagrelor and aspirin was administered prior to angiography. The patient was noted to have 90% ulcerated plaque of the proximal left anterior descending artery (Figure 3), which was successfully treated with a 3.0 x 18 mm XIENCE Sierra drug-eluting stent (Abbott, Abbott Park, IL, USA), reducing the 90% lesion to 0%, with post-PCI TIMI III flow noted (Figure 4). His anginal symptoms resolved after percutaneous coronary intervention (PCI). EKG abnormalities resolved with troponin peaking at 90.431 after the PCI. Medical therapy was optimized for CAD and acute systolic dysfunction prior to discharge from the hospital.

## Discussion

In the subset of patients with VPR, diagnosis of STEMI can be challenging. Currently, there are no validated diagnostic criteria recognized for the diagnosis of STEMI on VPR [1,2]. It has been suggested that having diagnostic criteria for STEMI in patients with VPR will lead to early reperfusion and better clinical outcomes [2,4,5]. LBBB is not a rare finding in patients with myocardial infarction (MI); however, the proportion of patients with LBBB and chest pain who actually have MI varies between 13% and 32% [1,6-8]. In addition, almost one-half of patients with LBBB and MI do not have chest pain, which further emphasizes the importance of an accurate diagnostic tool [9].

**Figure 5:**
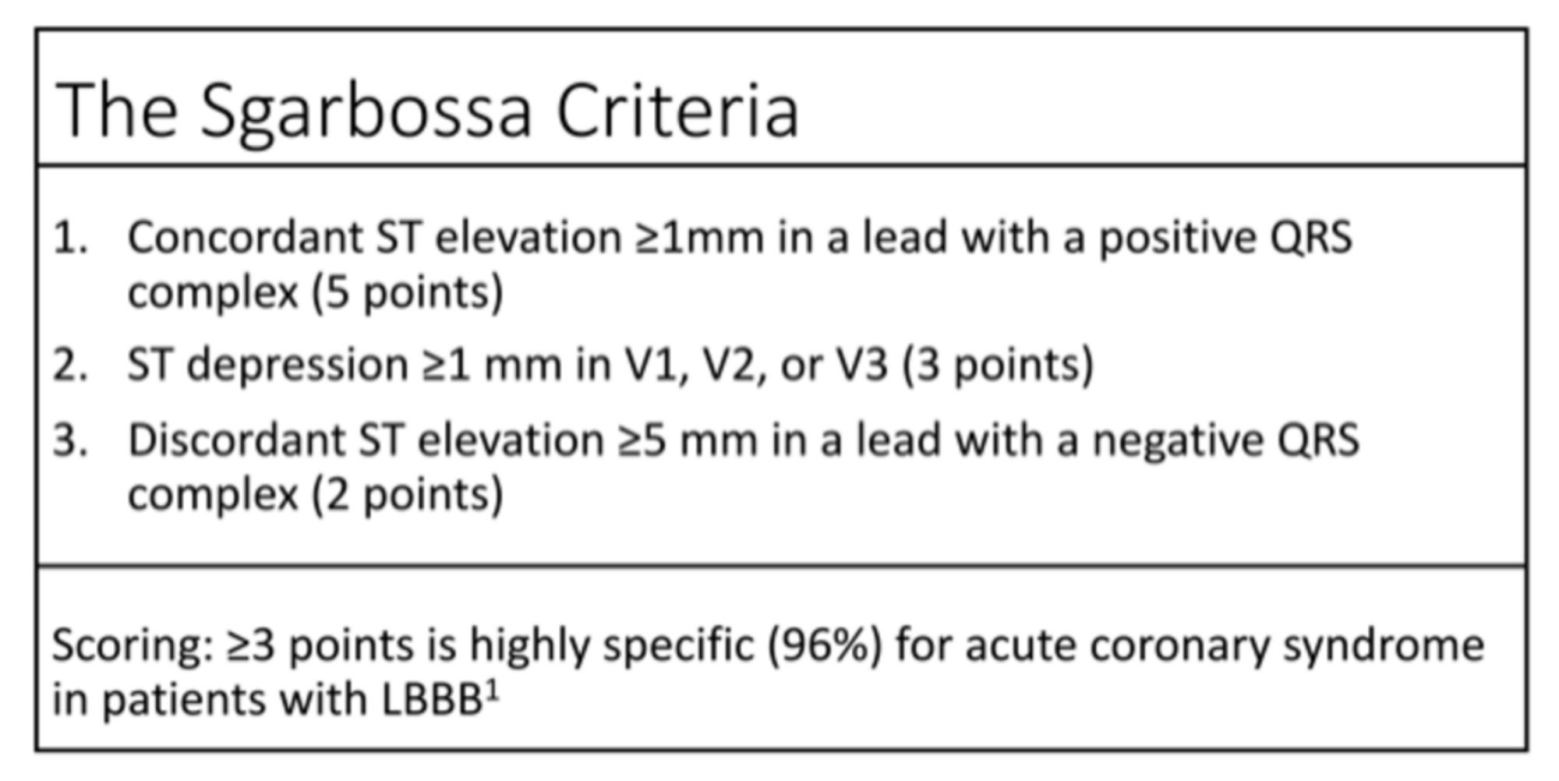
Commonly used Scarbossa criteria for the diagnosis of STEMI in the setting of LBBB. STEMI, ST-elevation myocardial infarction; LBBB, left bundle branch block

Previously described EKG criteria may be used to predict STEMI in patients with VPR and LBBB. The Sgarbossa criteria from the GUSTO-1 (Global Utilization of t-PA and Streptokinase for Occluded Coronary Arteries) trial have been shown to be highly specific for acute STEMI in the setting of known LBBB1 (Figure 5). A higher Sgarbossa score is correlated to increased mortality after STEMI and increased severity of MI [10]. The dilemma is that only 0.1% patients in GUSTO-1 trial had VPR, meaning there are insufficient data to extrapolate the results to VPR patients [1]. The Sgarbossa criteria can be used alongside the Smith-modified Sgarbossa criteria, which takes into account discordant STE with an amplitude of ≥25% of the depth of the preceding S-wave [1,11]. It remains controversial how effective these criteria are in the setting of VPR. Future studies should be directed at patients meeting the STEMI criteria on VPRs and the use of/utility of the aforementioned criteria in the diagnosis of acute STEMI.

## Conclusions

This case study illustrates how the Sgarbossa criteria along with good clinical acumen can potentially be applied to VPR and lead to the diagnosis of STEMI. In addition to EKG changes, the patient’s presenting symptoms, laboratory abnormalities, and echocardiogram were all taken into account to reach the diagnosis of STEMI.
